# P-608. Clinical Profile of GSK's Pentavalent Meningococcal ABCWY Vaccine: An Overview

**DOI:** 10.1093/ofid/ofae631.806

**Published:** 2025-01-29

**Authors:** Daniela Toneatto, Maria Lattanzi, Ellen Ypma, Karen O’Brien, Steven Rubin, Laura Tomasi Cont, Paola Pedotti, Woo-Yun Sohn

**Affiliations:** GSK, Sienna, Toscana, Italy; GSK, Sienna, Toscana, Italy; GSK, Sienna, Toscana, Italy; GSK, Sienna, Toscana, Italy; GSK, Sienna, Toscana, Italy; GSK, Sienna, Toscana, Italy; GSK, Sienna, Toscana, Italy; GSK, Sienna, Toscana, Italy

## Abstract

**Background:**

GSK’s MenABCWY vaccine was developed to provide broad protection against invasive meningococcal disease by combining the antigenic components of the licensed 4-component meningococcal serogroup B vaccine (MenB-4C) and serogroups A,C,W,Y vaccine (MenACWY-CRM).

**Methods:**

An overview of the MenABCWY vaccine’s profile was derived from its clinical development program of 12 controlled studies,^1-10^ which enrolled over 7000 adolescents and adults in 13 countries.

**Results:**

For serogroup B (MenB), non-inferiority of MenABCWY (2 doses, 6 months apart) to MenB-4C (2 doses, 2 months apart) was demonstrated in a phase 3 study^9^ in terms of vaccine-induced bactericidal immune responses measured by endogenous complement human serum bactericidal antibody (enc-hSBA) assay. The enc-hSBA assay uses each vaccinee’s serum as source of complement and tests a panel of 110 MenB strains representing 95% of disease-causing isolates in the US and ∼89% of global circulating strains,^11^ and is thereby used to assess immunological vaccine effectiveness (iVE) in clinical studies, in conditions close to real-world settings. MenABCWY iVE was 78% (95% CI: 77–79) against the panel of 110 MenB strains; serum from 84% of participants killed ≥ 70% of strains.^9^ For serogroups A,C,W,Y, non-inferiority of MenABCWY (2 doses, 6 months apart) to MenACWY-CRM (1 dose) was demonstrated in phase 3 studies in MenACWY-primed^10^ and -naïve^9^ participants, with robust immune responses against each serogroup.^9,10^ Phase 2 data for all 5 serogroups show 2-year and 4-year antibody persistence^5,8^ and anamnestic booster responses^2,5,8^ at 2 years after primary MenABCWY vaccination, and similar immune responses with MenABCWY 0-6 and 0-11 months schedules.^8^ In phase 3, 1 MenABCWY dose was non-inferior to 1 MenACWY-CRM dose as booster in participants primed with MenACWY ≥ 4 years previously.^10^ The safety and reactogenicity profile of MenABCWY was favorable overall and consistent with that of MenB-4C.^1-3,8,9^

**Conclusion:**

GSK’s combined MenABCWY vaccine is immunogenic, with demonstrated non-inferiority to MenB-4C and MenACWY-CRM vaccines, and a safety profile similar to that of MenB-4C.

FUNDING: GSK

**Disclosures:**

**Daniela Toneatto, MD**, GSK: Employee|GSK: Stocks/Bonds (Public Company) **Maria Lattanzi, MD, PhD**, GSK: Employee|GSK: Stocks/Bonds (Private Company) **Ellen Ypma, Msc**, GSK: Employee **Karen O’Brien, PhD**, GSK: Employee **Steven Rubin, PhD**, GSK: Employee **Laura Tomasi Cont, PhD**, GSK: Employee|GSK: Stocks/Bonds (Private Company) **Paola Pedotti, PhD**, GSK: Employee|GSK: Stocks/Bonds (Private Company) **Woo-Yun Sohn, MD**, GSK: Employed by GSK|GSK: Stocks/Bonds (Private Company)

